# Characterization of expressed sequence tags from developing fibers of *Gossypium barbadense* and evaluation of insertion-deletion variation in tetraploid cultivated cotton species

**DOI:** 10.1186/1471-2164-14-170

**Published:** 2013-03-13

**Authors:** Yuanda Lv, Liang Zhao, Xiaoyang Xu, Lei Wang, Cheng Wang, Tianzhen Zhang, Wangzhen Guo

**Affiliations:** 1 National Key Laboratory of Crop Genetics & Germplasm Enhancement, Cotton Research Institute, Nanjing Agricultural University, Nanjing, 210095, China

## Abstract

**Background:**

Cotton is the leading fiber crop worldwide. *Gossypium barbadense* is an important species of cotton because of its extra-long staple fibers with superior luster and silkiness. However, a systematic analysis and utilization of cDNA sequences from *G. barbadense* fiber development remains understudied.

**Results:**

A total of 21,079 high quality sequences were generated from two non-normalized cDNA libraries prepared by using a mixture of *G. barbadense* Hai7124 fibers and ovules. After assembly processing, a set of 8,653 unigenes were obtained. Of those, 7,786 were matched to known proteins and 7,316 were assigned to functional categories. The molecular functions of these unigenes were mostly related to binding and catalytic activity, and carbohydrate, amino acid, and energy metabolisms were major contributors among the subsets of metabolism. Sequences comparison between *G. barbadense* and *G. hirsutum* revealed that 8,245 unigenes from *G. barbadense* were detected the similarity with those released publicly in *G. hirsutum*, however, the remaining 408 sequences had no hits against *G. hirsutum* unigenes database. Furthermore, 13,275 putative ESTs InDels loci involved in the orthologous and/or homoeologous differences between/within *G. barbadense* and *G. hirsutum* were discovered by *in silico* analyses, and 2,160 InDel markers were developed by ESTs with more than five insertions or deletions. By gel electrophoresis combined with sequencing verification, 71.11% candidate InDel loci were reconfirmed orthologous and/or homoeologous loci polymorphisms using *G. hirsutum* acc TM-1 and *G. barbadense* cv Hai7124. Blastx result showed among 2,160 InDel loci, 81 with significant function similarity with known genes associated with secondary wall synthesis process, indicating the important roles in fiber quality in tetraploid cultivated cotton species.

**Conclusion:**

Sequence comparisons and InDel markers development will lay the groundwork for promoting the identification of genes related to superior agronomic traits, genetic differentiation and comparative genomic studies between *G. hirsutum* and *G. barbadense*.

## Background

Cotton (*Gossypium* spp.) is the leading fiber crop worldwide. There are four cultivated cotton species, two diploids from Africa-Asia, *G. herbaceum* L. (Gher, A_1_ genome) and *Gossypium arboreum* L. (Ga, A_2_ genome), and two tetraploids from Americas, *G. hirsutum* L. (Gh, AD_1_ genome) and *G. barbadense* L. (Gb, AD_2_ genome). At present, *G. hirsutum* is the most widely cultivated cotton species, accounting for more than 95% of the world cotton production (National Cotton Council, 2012, http://www.cotton.org/econ/cropinfo/index.cfm), followed by *G. barbadense* (accounting for 2 ~ 3%) and *G. arboreum* (accounting for 1 ~ 2%), while *G. herbaceum* is rarely cultivated. *G. hirsutum* has the characteristics of high yield, broad adaptation, and medium fiber quality. *G. barbadense* typically has a longer growing period, matures later, and produces smaller bolls that give a yield significantly lower than that of *G. hirsutum*. In spite of these drawbacks, however, *G. barbadense* possesses superior fiber properties, which makes it an important raw material for high-grade or special cotton fiber textiles [1].

Recently, a number of genome resources have been developed from the genus *Gossypium* including the construction of high-density tetraploid cotton genetic linkage maps [2-7], construction of large-insert BAC libraries [8,9], and analyses of expressed sequence tags (EST) related to fiber development [10,11]. Of these, EST analysis is not only the most efficient approach for gene discovery, but also an effective approach for the development of polymorphic DNA markers. As of Jan. 20, 2012, approximately 414,265 cotton EST sequences are available in Genbank ESTs database (http://www.ncbi.nlm.nih.gov/dbEST/). Among them, 297,214 ESTs were from *G. hirsutum*, 63,577 from *G. raimondii*, 41,781 from *G. arboreum*, while only 11,446 and 247 were from *G. barbadense* and *G. herbaceum*, respectively. Compared with great amount of EST resources from *G. hirsutum*, ESTs for *G. barbadense* are relatively scarce and have hindered the exploration of its economic importance.

Large scale transcript sequences offer an efficient resource for targeted marker development. In cotton, SSR markers have been mined widely based on existing sequences data from different cotton species [2,12-17], and applied widely in characterizing variations of genes, genome-wide mapping [18-21], and as a tool for marker-assisted selection [22-26]. In addition to SSR loci, the distribution of insertion-deletion (InDel) and single nucleotide polymorphisms (SNPs) variations are more widespread in the whole genome. Recently, InDel and SNPs are increasing in their application in studies of cotton genomics [27-29]. As an application, 223 SNP markers were mapped in 186 recombinant inbred lines from a cross between TM-1 and 3–79 [28]. Information from these large-scale markers will serve as a foundation for constructing high-density genetic maps, cloning and mapping of important genes, improving gene prediction and annotation, and elucidating the interspecific divergence in different cotton species.

In this study, we constructed two non-normalized cDNA libraries from fibers and ovules mixtures at −3 to 5 days post-anthesis (DPA) and fibers at 6 to 24 DPA, for efficient generation of unique ESTs from *G. barbadense* cv. Hai7124. In total, 21,079 ESTs were generated by a large-scale 5^′^ end single-pass sequencing of randomly picked cDNA clones from the two libraries. Upon adding the released 11,446 *G. barbadense* EST sequences in the National Center for Biotechnology Information (NCBI), EST assembly for *G. barbadense* was performed and functional categories of unigenes were assigned. Further, by meta-analysis of fiber ESTs between *G. barbadense* and *G. hirsutum*, some putative *G. barbadense*-specific expressed fiber genes and InDel loci of orthologous or homoeologous variation between/within *G. barbadense* and *G. hirsutum* were developed, which put the foundation for promoting the identification of genes related to superior agronomic traits, genetic differentiation and comparative genomic studies between *G. hirsutum* and *G. barbadense*.

## Results

### Generation of *G. barbadense* fiber ESTs

Two high-quality *G. barbadense* cotton fiber cDNA libraries were constructed using Hai7124 developing fiber tissues, one for −3 to 5 DPA (named as GB1 library) and the other for 6 to 24 DPA (named as GB2 library) respectively. Once each library was constructed, the lengths of inserts were identified by amplifying randomly 100 clones with universal T7 primers. The results showed that most of the inserts were among 0.5-1.2 kb, with an average length of 0.7 kb for the GB1 library and 0.8 kb for the GB2 library. Based on this, 22,329 cDNA clones (12,040 from GB1 library and 10,289 from GB2 library) were randomly isolated and sequenced to generate ESTs from the 5^′^ end. After the removal of low quality sequences below the Q20 threshold, short sequences less than 100 bp in length, and those with vector sequences and poly-A tails, 21,079 high-quality sequences with scores ranging from 40 to 60 were obtained for further analysis. Most of the sequences ranged from 500 to 900 bp, with an average sequence length of 652 bp with the longest EST at 877 bp. All high quality sequences have been deposited in the dbEST division of GenBank under the accession numbers JK790134 to JK811212.

### Assembly and function annotation of *G. barbadense* fiber ESTs

To reduce the redundancy and facilitate the process of gene annotation and mapping, all *G. barbadense* EST sequences available in Genbank and herein were combined for EST processing and assembly analysis. Of 32,525 ESTs, 4,938 were singletons and the remaining were assembled into 3,715 contigs, yielding 8,653 unigenes for further analysis. The average sequence length of unigenes was 712 bp, and the longest unigene was 2,331 bp. We subjected 8,653 unigenes to Blastx against the non-redundant (nr) database to identify their similarities with known proteins (Additional file 1: Table S1). In total, 7,786 (89.98%) sequences shared the high similarity with public protein sequences, of these, 7,224 (83.49%) matched known proteins and 562 (6.49%) were unknown or hypothetical proteins. The remaining 867 (10.02%) sequences had no similarities in the tested databases and might be either 3^′^ or 5^′^ untranslated regions (UTRs) of genes with too short of a coding sequence, or novel genes [30].

### Function classification and metabolic pathway analysis of unigenes

Of the 8,653 unigenes, 7,316 were mapped to the GO hierarchy with characterized biochemical and physiological functions using Gene ontology annotation (Additional file 1: Table S1). Of these, 6,348 were mapped to GO categories, and were involved with biological processes, molecular functions, and cellular components. At a second level, the majority of the GO terms were grouped into cellular process (32.30%) and metabolic process (29.89%) categories within biological processes, binding (43.56%) and catalytic activity (31.42%) categories within molecular functions, and cell part (28.79%), cell (28.79%), and organelle (22.55%) categories within cellular components.

**Figure 1:**
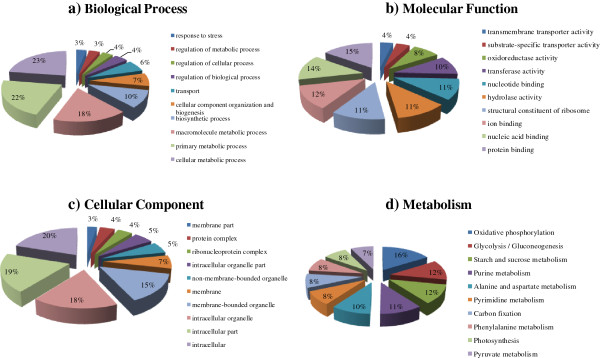
**Functional classifications for 8,653 unigenes with GO terms and KEGG terms (third level terms).** (**a**) Biological process; (**b**) Molecular function; (**c**) Cellular component; (**d**) Metabolism (KEGG). More detailed information is provided in Additional file 1: Table S1.

At a third level, the different categories were further mined. In biological processes (Figure 1a), the metabolic process was the most enriched as “cell metabolic process” ranked first, accounting for 16.08% of the unigenes involved with biological processes. “Primary metabolic processes” was second with 15.24%, and third was the “macromolecular metabolic processes” with 12.63% of the unigenes. Within the molecular function category (Figure 1b), the highly enriched GO terms in “binding” were distributed in the following way: 12.09% represented “protein binding”, 10.81% were “nucleic acid binding”, 9.84% were “ion binding”, and 8.73% were involved with “nucleotide binding”. The top three highly enriched GO terms in “catalytic activity” were 9.00% belonging to “hydrolase activity”, 7.90% on transferase activity, and 6.46% on oxidoreductase activity. Furthermore, the cellular component mostly took place in “intracellular” with 18.51%, “intracellular part” with 18.11% and “intracellular organelle” with 16.50% (Figure 1c).

In addition, by a comparison with the Kyoto Encyclopedia of Genes and Genomes database (KEGG), the metabolic-related enzymes encoded by 4,436 unigenes were also located in metabolic maps based on the KEGG pathway classification (Table 1, Additional file 2: Table S2). The metabolism category was the richest (2,192, 49.41%), followed by the categories of environmental information processing (415, 9.36%), cellular processes (294, 6.63%), genetic information processing (192, 4.33%), organismal systems (328, 7.39%) and metabolic pathways related to human diseases (1,015, 22.88%). In the category of metabolism, the mapped enzymes were mostly involved in carbohydrate metabolism (609 clusters), amino acid metabolism (330 clusters), and energy metabolism (304 clusters). In the category of cellular process, 62.59% were related to cell growth and death (184 clusters). In the category of organismal systems, the endocrine system (164, 26.45%), and immune system (124, 20.00%) were major contributors. In the category of environmental information processing, 99.76% of unigenes were involved in signal transduction. In the category of genetic information processing, folding, sorting and degradation was the majority (113, 58.85% of the category), followed by transcription (35, 18.23%) and replication and repair (31, 16.15%). At a third level, the mappings of metabolism associated with oxidative phosphorylation (16%), glycolysis/gluconeogenesis (12%), and starch and sucrose metabolism (12%) were further tagged (Figure 1d).

**Table 1:** Summary for the metabolic analysis of unigenes (second level)

		
**1 Metabolism**	**2192**	**49.41%**
Carbohydrate metabolism	609	13.73%
Energy metabolism	304	6.85%
Lipid metabolism	188	4.24%
Nucleotide metabolism	170	3.83%
Amino acid metabolism	330	7.44%
Metabolism of other amino acids	185	4.17%
Glycan biosynthesis and metabolism	13	0.29%
Metabolism of cofactors and vitamins	151	3.40%
Metabolism of terpenoids and polyketides	67	1.51%
Biosynthesis of other secondary metabolites	91	2.05%
Xenobiotics biodegradation and metabolism	84	1.89%
**2 Genetic information processing**	**192**	**4.33%**
Transcription	35	0.79%
Translation	13	0.29%
Folding, sorting and degradation	113	2.55%
Replication and repair	31	0.70%
**3 Environmental information processing**	**415**	**9.36%**
Signal transduction	413	9.31%
Signaling molecules and interaction	2	0.05%
**4 Cellular processes**	**294**	**6.63%**
Transport and catabolism	2	0.05%
Cell motility	69	1.56%
Cell growth and death	184	4.15%
Cell communication	39	0.88%
**5 Organismal systems**	**328**	**7.39%**
Immune system	124	2.80%
Endocrine system	164	3.70%
Nervous system	19	0.43%
Sensory system	2	0.05%
Development	19	0.43%
**6 Human diseases**	**1015**	**22.88%**
Cancers	479	10.80%
Immune diseases	25	0.56%
Neurodegenerative diseases	350	7.89%
Endocrine and metabolic diseases	14	0.32%
Infectious diseases	147	3.31%

### Transcript abundance and highly enriched genes during fiber development

**Figure 2:**
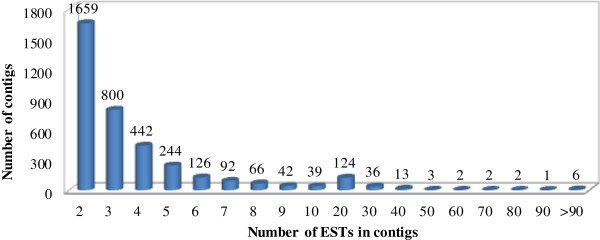
Distribution of contigs based on the number of assembled ESTs.

The level of representation in a cDNA library generally correlates with transcript abundance in non-normalized conditions. We assessed the distribution of contigs based on the number of assembled ESTs. Compared with singletons, most of the contigs consisted of 2 to 5 ESTs and a few were comprised of more than 100 ESTs (Figure 2). The top 10 most highly expressed unigenes and their annotations can be found in the Table 2, including non-specific lipid-transfer protein family, copper-zinc superoxide dismutase, E6, fiber protein (fb10), which was responsible for fiber development and growth in previous studies [31-35].

**Table 2:** Top 10 highly enriched genes in fiber developmental stages

**Unigene_ID**	**ESTs Num.**	**Seq description**	**Length**	**eValue**
Unigene0544	992	lipid transfer protein family	630	1.0E-52
Unigene3080	445	E6 protein	812	1.0E-38
Unigene2175	203	histone h3	744	1.0E-70
Unigene2382	178	copper-zinc superoxide dismutase	598	1.0E-71
Unigene1298	162	polyubiquitin	1157	1.0E-167
Unigene2429	146	fiber protein fb10	602	1.0E-55
Unigene1075	130	alpha tubulin 1	1656	0
Unigene0129	127	plasma membrane intrinsic protein	1054	1.0E-147
Unigene2564	96	arabinogalactan protein	1036	1.0E-89
Unigene0009	91	anthocyanidin reductase	1318	0
Unigene0343	90	vacuolar h^+^-atpase c subunit	848	1.0E-56
Unigene2530	78	metallothionein-like protein	559	1.0E-26
Unigene0786	74	xyloglucan endotransglycosylase	1172	1.0E-145
Unigene1516	69	ribosomal protein l9	889	1.0E-95
Unigene2082	66	alpha expansin	1181	1.0E-125
Unigene1627	65	60s acidic ribosomal protein p1	623	1.0E-19
Unigene1504	64	dihydroflavonol 4-reductase	1361	0
Unigene0270	58	ubq10 (polyubiquitin 10) protein binding	909	1.0E-118
Unigene0180	58	mads-box transcription factor	860	1.0E-120
Unigene0658	38	glyceraldehyde-3-phosphate dehydrogenase	1349	1.0E-170

### Comparisons with the *G. hirsutum* ESTs

ESTs data from *G. hirsutum* were downloaded from GenBank. Poly A/T sequences occurring at the end of ESTs and vectors were removed from original sequences. As a result, a set of cleaned ESTs generated from *G. hirsutum* was clustered and assembled using the CAP3 assembly program. The clustering of ESTs from *G. hirsutum* yielded 94,955 unigenes, with 25,290 contigs and 69,665 singletons, respectively.

To find putative specific expression genes in *G. barbadense*, 8,653 unigenes from *G. barbadense* were used to detect similarity with *G. hirsutum* unigenes using the TBlastx program with a significant similarity (E-value) threshold of 10^-10^. As a result, 8,245 unigenes from *G. barbadense* were detected the similarity with *G. hirsutum*. The remaining 408 sequences had no hits against the *G. hirsutum* unigenes database.

The putative 408 specific expression genes in *G. barbadense* were assigned functions by Blastx against the nr protein database. Of these, 213 sequences had one or more similarities with proteins in the non-redundant protein database, however, 195 sequences had no significant similarity. According to ESTs abundance, we also confirmed the expression specificity/predominance of these genes by selecting seven putative genes at random to analyze their expression patterns at 10 and 20 DPA in the fiber developmental process between *G. hirsutum* acc. TM-1 and *G. barbadense* cv. Hai7124 using RT-PCR method (Additional file 3: Figure S1). These putative *G. barbadense* specific expressed genes encoded mainly for the vacuolar H^+^-atpase c subunit, profiling, anthocyanidin synthase, proline-rich protein, kinetochore protein, etc. The detailed annotation information and abundance of specific expressed genes in *G. barbadense* were listed in supplementary Additional file 4: Table S3.

### Large-scale discovery and confirmation of InDels between/within *G. barbadense* and *G. hirsutum* ESTs

InDels can lead to differentiation of species, and small InDels were widely distributed in the different species. In the study, 297,214 *G. hirsutum* and 32,525 *G. barbadense* EST sequences were assigned as two distinct data sets for all possible InDels loci based on *in silico* PCR strategy. A set of primer pairs from *G. barbadense* EST sequences were run against the *G. hirsutum* EST sequences dataset based on *in silico* PCR analysis with a threshold less than 3 mismatch bases. In total, 28,426 InDel loci derived from 18,797 *G. barbadense* ESTs were identified using a custom InDel_pipeline.pl script. After redundancy analysis, 13,275 unique EST InDel loci were detected for further study. Of these, 6,502 contained only 1 bp InDel size, 4,613 with 2 to 4 bp, 1,125 with 5 to 10 bp, and 1,035 had more than 10 bp. The average length of the InDels was 21 bp, with the longest InDels up to 168 bp (Additional file 5: Table S4).

Based on *in silico* PCR analysis, 2,160 InDel markers were developed with InDel lengths > =5 bp (Additional file 6: Table S5). To verify the accuracy and efficiency of InDel markers, 90 randomly selected InDel primer pairs were synthesized for the polymorphisms detection between/within *G. barbadense* and *G. hirsutum* using two tetraploids, *G. hirsutum* acc. TM-1 and *G. barbadense* cv. Hai7124, and two diploids, *G. herbaceum* and *G. raimondii*.

**Figure 3:**
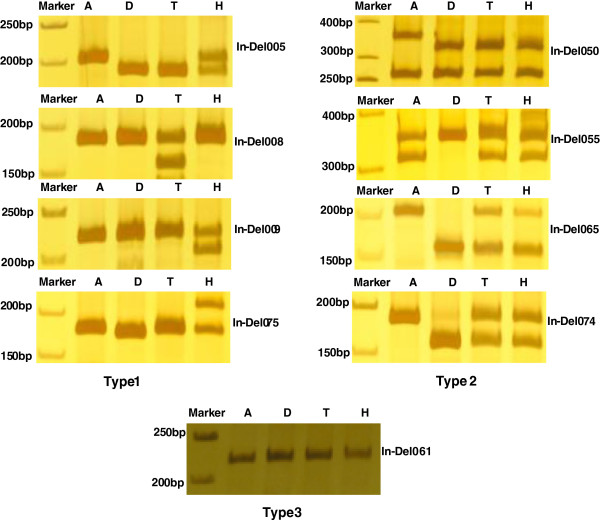
**Electropherogram and banding patterns of InDel primer pairs in four cotton species.** Type 1: Orthlogous loci polymorphisms between *G. barbadense* and *G. hirsutum*. Type 2: Homoeologous loci polymorphisms within *G. barbadense* and *G. hirsutum*. Type 3: Orthlogous and/or homoeologous loci monomorphisms between/within *G. barbadense* and *G. hirsutum*. Note: From left to right in electropherogram: M: marker; A: *G. herbaceum*; D: *G. raimondii*; T: *G. hirsutum* acc. TM-1; H: *G. barbadense* cv. Hai7124*.*

Of 90 randomly selected InDel loci, 64 primer pairs showed distinguishable orthologous and/or homoeologous polymorphisms (Figure 3), with 46.87% (from 30 InDel markers) orthologous polymorphisms (Type 1 in Figure 3) and 53.13% (from 34 InDel markers) homoeologous polymorphisms (Type 2 in Figure 3). In addition, 20 InDel primer pairs were unable to produce detectable polymorphic electrophoresis bands between *G. hirsutum* and *G. barbadense* (Type 3 in Figure 3), and 6 InDel primer pairs failed to amplify PCR products in tested cotton accessions. Amplification information for 90 InDels markers is included in the Electronic Supplementary Additional file 7: Table S6.

For confirming the accuracy of InDel loci in the transcriptional regions, the amplification products from 9 InDel primer pairs with different types (Figure 3) were individually recovered from polyacrylamide gels and sequenced. The length of all amplicons was equal to or greater than that expected from *G. barbadense*. Sequences alignment analysis from orthologous and/or homoeologous loci amplified by 8 InDel primer pairs (Type 1 and Type 2 in Figure 3), showed that, except for size difference in intron region, InDels from the corresponding exon regions were all confirmed the existence, just like the result of *in silico* analysis. To undetectable polymorphic electrophoresis bands from InDel061, sequencing analysis also showed the complete consistency of orthologous loci from four cotton species, indicating that the InDels from the transcriptional level might be due to alternative splicing events with no difference at the genome level. As for failure PCR amplification between *G. hirsutum* and *G. barbadense*, this might be related with the reason that the primer sequence from EST spanned the intron–exon boundary of the genome and need to be confirmed in the future.

### Insertion-deletion EST variation in tetraploid cultivated cotton species might be responsible for fiber quality

During fiber developmental stage, cellulose synthesis and secondary cell wall (SCW) thickening is one of the most important events for fiber quality. To further confirm whether insertion-deletion EST variation in tetraploid cultivated cotton species might be related to fiber quality difference between *G. hirsutum* and *G. barbadense*, 2,160 candidate InDel loci with InDel lengths > =5 bp were further assessed by blastx against TAIR (The Arabidopsis Information Resource) database (http://www.arabidopsis.org/index.jsp) to identify genes involved in secondary wall synthesis. As a result, 81 InDel loci with significant function similarity with known genes associated with secondary cell wall synthesis process of *Arabidopsis thaliana* trichomes (Additional file 8: Table S7). Of them, 72 were targeted to encode the cellulose synthase related genes, such as cellulose synthase, ras-related GTP-binding family, germin-like protein, beta-1,3-glucanase, glycosyl transferase (GTs), fasciclin-like arabinogalactan (FLA), chitinase-like protein (CTL), arabinogalactan (AGP), and sucrose synthase (Sus). Other nine were targeted to encode the transcriptional factor related genes, such as myb-related protein, homeodomain protein, and zinc finger protein.

In these predicted genes, some family genes have been identified as a major role in cotton fiber secondary wall synthesis, such as *GhCesA* (cellulose synthase catalytic subunit) [36-38], *GhFLA* (fasciclin-like arabinogalactan)[39,40], *GhAGP* (Arabinogalactan protein) [41], *Rac13*[42], *GhCTL* (chitinase-like) [43], and *Sus* (sucrose synthase) [44]. The insertion-deletion EST variation of these key genes related to fiber secondary cell wall synthesis might play the important roles in fiber quality in tetraploid cultivated cotton species.

## Discussion

### *G. barbadense* ESTs - indispensable cotton breeding sequences resource

*G. barbadense* has been used more commonly as a gene donor for the high-quality cotton fiber trait. The hybrids produced with *G. hirsutum* are expected to have the desirable characteristics including a high yield, exceptional fiber length, fineness and strength. However, the lack of *G. barbadense* genomic resources has seriously hampered the exploration of modern functional genomic approaches for selective breeding purposes.

Expressed Sequence Tag (EST) sequencing strategies are efficient in identifying a large number of genes expressed in a given tissue, which are particularly relevant when no genomic data are available [45]. Prior to this publication, only one *G. barbadense* dataset was publicly released, which included 11,446 ESTs from *G. barbadense* cv. 3–79 fiber tissues [46]. In our study, 21,079 high quality sequences were generated from two non-normalized cDNA libraries prepared by using a mixture of Hai7124 fibers and ovules at −3 to 5 days post-anthesis (DPA) and fibers at 6 to 24 DPA. The assembly resulted in approximately 8,653 unigenes, involving 3,715 contigs and 4,938 singletons. About 83% of contigs and singletons reported here were matched with high confidence to the nr protein database. These sets of ESTs are a large contribution to enhance the number of *G. barbadense* ESTs available in public databases.

In addition, a high level of representation in a cDNA library generally correlates with transcript abundance in non-normalized conditions. Abundance analysis revealed that large clusters existed (high expression), such as lipid-transfer protein precursors, copper-zinc superoxide dismutase, E6, and arabinogalactan protein, which was in agreement with many previously reported cotton fiber key genes. Orford and Timmis (2000) suggested lipid transfer protein gene family might play an important role in fiber development [47]. John et al. (1996) described the isolation of the E6 gene that was expressed preferentially in cotton fiber tissues on the 15^th^ and 24^th^ days after flowering [33]. Superoxide dismutases, commonly found in all oxygen-consuming organisms, are a class of metal enzymes, which can remove superoxide anion radicals. Hu et al. (2007) reported that the copper/zinc superoxide dismutase mRNA expressing level showed regular changing in the fiber developmental process [32]. Other highly expressed genes such as anthocyanidin reductase [35], pectate lyase [34], arabinogalactan protein [41] and fiber protein fb10 also were found to play an important role in the cotton fiber development.

### Comparative analysis between *G. hirsutum* and *G. barbadense*

Comparative analysis of ESTs from fiber development between *G. hirsutum* and *G. barbadense* will facilitate the identification of genes related to the important agronomic traits such as fiber growth and development. TblastX comparisons between *G. barbadense* fiber sequences and those from *G. hirsutum* released publicly in GenBank revealed that 8,245 unigenes (95.3%) from *G. barbadense* were detected as similarities with those from *G. hirsutum.* The remaining 408 sequences had no hits against the *G. hirsutum* unigenes database. Though the E-Value setting, UTRs regions' interference, and insufficient coverage of *G. hirsutum* ESTs database might inevitably cause certain false positives in the process of similarity analyses, the expression predominance of selected seven unigenes in *G. barbadense* was confirmed experimentally by comparing 10 and 20 DPA fiber tissues between *G. hirsutum* acc. TM-1 and *G. barbadense* cv. Hai7124. Based on the fact that *G. barbadense* possesses superior fiber quality, key genes related to fiber quality in *G. barbadense* may be exploited in cotton molecular breeding programs for improving fiber quality. The predominantly expressed sequences found here will provide an insight into exploring interspecific fiber quality divergence between *G. barbadense* and *G. hirsutum.* Further function confirmation of these sequences will be verified by experimental analyses.

### Elite genes can be mined effectively by large-scale discovery of InDels between *G. barbadense* and *G. hirsutum*

Currently, there is an increasing focus on polymorphisms of short insertions and deletions (InDels) types in genomic research of model species such as humans [48], mice [49] and rice [50]. InDels have been recognized as an abundant source of genetic markers that are widely spread across the genome, and can be genotyped with simple size separation based on PCR amplification and gel electrophoresis analyses. Thus, the InDel molecular markers were highly informative sequence-based markers suitable for high-density map construction, genome-wide association studies, genomic selection, and alignment of the whole genome sequence information.

In allopolyploids, mutational variation may arise between homologous sequences within individual subgenomes and between homoeologous sequences among subgenomes, in addition to paralogous variation between duplicated gene copies [51]. When information regarding genomic sequences is not rich, InDels polymorphism of tetraploid cotton species obtained by computational analysis might include both homologous and homoeologous loci variation. Nevertheless, mining these differences will be useful for verifying the function of the target genes and elucidating evolution in polyploid. In the study, we first reported the feasibility of using large-scale ESTs sequence data from different cotton species to extract putative insertion and deletion polymorphisms. 13,275 InDel loci were identified between *G. barbadense* and *G. hirsutum*, and 2,160 potential Gh-Gb InDel markers were further developed for genotyping and evolutionary research. Further, the InDel-derived ESTs/genes related to fiber growth and development was detected. In the paper, we found some genes involved in secondary cell wall synthesis process with potential EST InDel loci, such as those encoding cellulose synthase, sucrose synthase, beta-1,3-glucanase, glycosyl transferase, fasciclin-like arabinogalactan protein, arabinogalactan protein, chitinase-like protein, and other transcriptional factor genes involved in secondary cell wall synthesis. In *Arabidopsis*, InDel mutations of cellulose synthase genes in secondary cell wall synthesis had been known to cause structural weaknesses in vascular bundles presumably due to cellulose deficiency [52,53]. So, the deep research of these genes will accelerate our understanding to fiber growth and development process in cotton.

Due to the fact that all InDel loci were discovered based on transcriptional level and all *G. barbadense* ESTs were collected from different stages of fiber development, Gh-Gb InDels enriched many genes related to cotton fiber development, which could greatly promote their deep research for structural and expressional differences related to the fiber development processes in *G. barbadense* and *G. hirsutum*. By randomly selecting 90 InDels primer pairs to conduct PCR analysis of genomic DNA, with the sequencing confirmation from 9 InDel loci in four cotton species, more than 70% InDel polymorphic loci (64/90 = 71.11%) were detected effectively. From the result, not only homologous and homoeologous loci difference were found, but also some evolutionary events could be inferred. The diversity among the allotetraploid cottons such as orthologous and/or homoeologous polymorphisms could be traced back to ancient diploid ancestors by banding patterns analysis, which indicated the independent evolution or different degrees of colonization by comparing *G. barbadense* and *G. hirsutum*, with two diploids, *G. herbaceum* and *G. raimondii*, as controls [4].

Taken together, InDel molecular detection provides a new tool for effectively mining genes related to superior agronomic traits, and selecting appropriate *G. barbadense* or *G. hirsutum* germplasm in cotton breeding. It also offers a novel model for the study of the origin, evolution, and genetic differentiation of *G. barbadense* and *G. hirsutum* and their adaptation to various environmental changes.

## Conclusion

Transcriptome analysis from different tissues and organs provides the basis for functional genomics research. In the present work, we constructed two *G. barbadense* fiber cDNA libraries and obtained 21,079 high-quality sequences. A systematic analysis and utilization of ESTs was further performed including assembling, annotation, GO classification and comparative analysis with *G. hirsutum*. The resulting dataset yielded nearly 8,653 putative unigenes, from which over 80% had similarities with publicly available proteins. Furthermore, putative ESTs InDels loci involved in the orthologous and/or homoeologous difference between/within *G. barbadense* and *G. hirsutum* were discovered by *in silico* analysis and confirmed by experimental analysis. The large-scale *G. barbadense* ESTs in the study were a significant contribution for public *G. barbadense* ESTs databases, and either expression or candidate EST InDel difference will provide a new tool for effectively mining genes related to superior agronomic traits. These data will provide a solid foundation for molecular breeding, functional genomics studies, and comparative genomics analysis in *Gossypium*. With the continuous growth of sequence information from non-model organisms such as cotton, we suggest that InDels will be a crucial source for next-generation mapping of key genes in these accessions.

## Methods

### Plant materials

*G. barbadense* cv. Hai7124, a commercial Sea-island *Verticillium-*resistant cultivar, was planted under standard field conditions at Jiangpu Breeding Station, Nanjing Agricultural University, Jiangsu Province, China, using normal farming practices in 2008. All necessary permits were obtained for the described field studies from Nanjing Agricultural University. Developing ovules of Hai7124 were excised from each boll at −3, 0, 3, 5, 6, 9, 12, 15, 18, 21, and 24 day post-anthesis (DPA) and used for RNA extraction. Of these, the samples at −3 to 5 DPA were collected from mixture of fibers and ovules, and the samples from 6 to 24 DPA were derived from fiber cells dissected from the ovules. All harvested plant materials were immediately frozen in liquid nitrogen and stored at −70°C.

### Construction of cDNA libraries and generation of ESTs

Total RNA from different fiber developmental stages was extracted using the CTAB-sour phenol extraction method [54]. Poly (A) + mRNA was purified from total RNA using an mRNA purification kit (Qiagen, Dusseldorf, Germany). A cDNA library was made using a smart™ cDNA library construction kit (Clontech Laboratories, Inc. Mountain View, USA, http://www.clontech.com). Automated DNA sequencing of >10,000 random cDNAs for each library from the 5^′^ -termini using universal T7 primers was performed with a Big Dye Terminator sequencing kit using Applied Biosystems (ABI) 3730 automated sequencers (Life Technologies Co., California, USA, http://www.lifetechnologies.com).

### EST processing and assembly

All *G. barbadense* EST sequences from NCBI and produced here were combined for EST processing and assembly analysis. The chromatogram traces were performed by Phred [55] for base-calling and poor quality segment trimming. A Phred quality score of 20 (corresponding to an error probability of 1%) was used for trimming the sequence based on quality, in order to retain a high quality sequence. The sequences that passed quality trimming were further masked vector and adapter sequences using the program Cross_Match [55] from the NCBI Univec database (ftp://ftp.ncbi.nih.gov/pub/UniVec/) with the following parameters: minmatch 20, minscore 20. PolyA tails and the “X” character were also removed by the EST_trimmer.pl script (http://pgrc.ipk-gatersleben.de/misa/download/est_trimmer.pl). After processing, sequences less than 100 bp were excluded from the analysis. ESTs larger than 100 bp length and Q20 quality after removing vectors, adapters, and ploy-A tails were performed to generate a unigene set using Cap3 [56] with the minimum overlapping length parameters >45 bp and overlapping identity percentage >90%. Cap3 assembly results were parsed using cap3_extractor.py script to identify those genes with the highest transcription abundance. All contigs and singletons were used for the further annotation.

### Function annotation and classification

The unigenes (contigs and singletons) were subjected to a similarity analysis using Blastx [57] against the NCBI nr (non-redundant) protein database. Blastx were performed at expectation value of 1e-05 to filter out nonspecific high-scoring segment pairs respectively.

The set of unigenes were submitted for GO (Gene ontology) [58] annotation to the Blast2GO program [59] with the default parameters. The program extracted the GO terms associated with homologies identified with BLAST and returned a list of GO annotations represented as hierarchical categories of increasing specificity.

Unigenes were assigned into metabolic pathways with the tools supplied by the Kyoto Encyclopedia of Genes and Genomes (KEGG) [60]. The unigenes were processed using the bi-directional “best hit” method (forward and reverse reads) to assign orthologs. KAAS (KEGG Automatic Annotation Server, http://www.genome.jp/kegg/kaas/) provided a functional annotation of putative genes by Blast comparisons against the KEGG GENES database. The output included KO (KEGG Orthology) assignments and automatically generated KEGG pathways.

### Comparisons to the *G. hirsutum* ESTs

For comparative analysis of fiber cDNAs between *G. barbadense* and *G. hirsutum*, 297,214 ESTs from *G. hirsutum* released in NCBI were downloaded in FASTA format and saved on a local computer. TBlastx program was used to screen for differential genes between *G. barbadense* and *G. hirsutum* with an expectation value of 1e-10. Differential genes then were assigned a putative function using Blastx against the nr protein database.

### Large-scale discovery of InDels between/within *G. barbadense* and *G. hirsutum* ESTs

As of Jan. 20, 2012, approximately 414,271 cotton EST sequences were available in Genbank ESTs database (http://www.ncbi.nlm.nih.gov/dbEST/). Of them, 329,749 ESTs from the two tetraploid cultivated cotton species, with 297,214 from *G. hirsutum* and 32,525 from *G. barbadense* (11,446 publically available in Genbank, 21,079 from our study), were assigned respectively as two distinct data sets for mining all possible InDels loci. *In silico* (virtual) PCR strategy was used to predict the possible InDel difference among orthologous and/or homoelogous loci between/within *G. barbadense* and *G. hirsutum*. In detail, each *G. barbadense* EST was first randomly cut into several segments about 300 bp for designing nested PCR primer pairs, which ensured the amplified regions to cover the whole EST sequence. Then, the set of primer pairs from *G. barbadense* EST sequences were run against the *G. hirsutum* EST sequences dataset based on *in silico* PCR analysis with a threshold less than 3 mismatch bases. At last, PCR products at given region with different size were used for mining potential InDel difference among orthologous and/or homoelogous loci between/within *G. barbadense* and *G. hirsutum*. As a result, InDel size larger than 5 bp was preferentially selected to confirm the reality by the gel electrophoresis combined with sequencing analysis, and homology analysis against previous reported genes associated with secondary wall synthesis of *Arabidopsis thaliana* trichomes [61-65] were performed by querying The Arabidopsis Information Resource (TAIR) database using Blastx alignment program by an E-value cutoff of 1e-5.

Forward and reverse flanking primer pairs based on *G. barbadense* ESTs were designed using Primer3 [66] by running the software in a batch mode. The primers varied in length from 18 to 20 bp (the optimal length 20 bp), with GC contents varying between 45% and 65% (50% GC content optimal). The lengths for target amplicon varied uniformly from 100 to 250 bp, and melting temperatures ranged from 57°C to 62°C with an optimal temperature of 58°C. All InDel primer pairs were synthesized by GenScript Inc. (Nanjing, China).

Hai7124 (*G. barbadense*) and TM-1 (genetic standard line in *G. hirsutum*) were used as parents for detecting orthologous and/or homoelogous loci polymorphisms between/within *G. barbadense* and *G. hirsutum*, with the two diploid progenitors, *G. herbaceum* and *G. raimondii*, as controls. The genomic DNA extraction followed that of Paterson et al. [67]. The InDels were amplified by a PTC-225 machine (MJ Research, USA), and gel electrophoresis of the amplicon was performed according to the methods described by Zhang et al. (2000) [68]. After *in silico* PCR analysis and gel electrophoresis confirmation, the accuracy of InDel loci was further evaluated by selecting at random the amplification products to sequence. For sequencing analysis, standard PCR reactions were performed using High-fidelity ExTaq DNA polymerase (TaKaRa Biotechnology Dalian Co., Ltd., China). The PCR products were cloned into the pMD18-T Vector (TaKaRa) according to the manufacturer’s instructions and sequenced from plasmid DNA templates. At least three positive clones were chosen to sequence. Sequencing was performed by GenScript Inc. (Nanjing, China).

## Competing interests

The authors declare that they have no competing interests.

## Authors’ contributions

Experiments were designed by WZG with suggestions from TZZ. Experiments were performed by YDL, LZ and LW. Bioinformatics analyses were performed by YDL and CW. LZ and XYX carried out experimental confirmation. YDL and WZG drafted the manuscript, and WZG revised the manuscript. All authors read and approved the final manuscript.

## Supplementary Material

Additional file 1: Table S1 Functional annotation and classification summary for 8653 unigenes. Note: These unigenes were submitted to nr (non-redundant) protein database and assigned with GO (Gene Ontology) and KO (KEGG) terms. Click here for file

Additional file 2: Table S2 Statistic analysis of KEGG classification with corresponding metabolism maps. Note: KO (KEGG) annotation and metabolism maps were performed with BLAST2GO. Classification showed in second level and third level. Click here for file

Additional file 3: Figure S1 Experimental verification of candidate unigenes expressed specifically in G. barbadense using RT-PCR. Click here for file

Additional file 4: Table S3 Function annotation of 408 putative G. barbadense -specific unigenes. Note: 408 unigenes without significant match to any sequence in the current *G. hirsutum* EST databases were identified and finished function annotation against nr (non-redundant) protein database. Click here for file

Additional file 5: Table S4 Summary for putative Gb-Gh Indel loci by in silic analysis. Notes: The information on insert size both in *G. barbadense* (Gb) and *G. hirsutum* (Gh), and size range was supplied in the table. Click here for file

Additional file 6: Table S5 2,160 Indel markers developed by using InDel > =5 bp EST sequences between G. barbadense and G. hirsutum. Click here for file

Additional file 7: Table S6 PCR amplification and banding patterns of randomly selected 90 Indel markers. Note: 1: No difference in tested accessions; 3: showed orthologous and/or homoeologous loci polymorphism in tested accessions. Click here for file

Additional file 8: Table S7 Function identification related to secondary wall synthesis using sequences detected InDels by tblastx analysis with databases from Arabidopsis. Click here for file
