# Supplementary material for: Characterization of expressed sequence tags from developing fibers of Gossypium barbadense and evaluation of insertion-deletion variation in tetraploid cultivated cotton species
Source: BMC Genomics. 2013 Mar 13;14:170. doi: 10.1186/1471-2164-14-170 (PMC3600364; doi:10.1186/1471-2164-14-170)
Supplement: Additional file 3: Figure S1 — Experimental verification of candidate unigenes expressed specifically in G. barbadense using RT-PCR. [file 1471-2164-14-170-S3.doc]

**
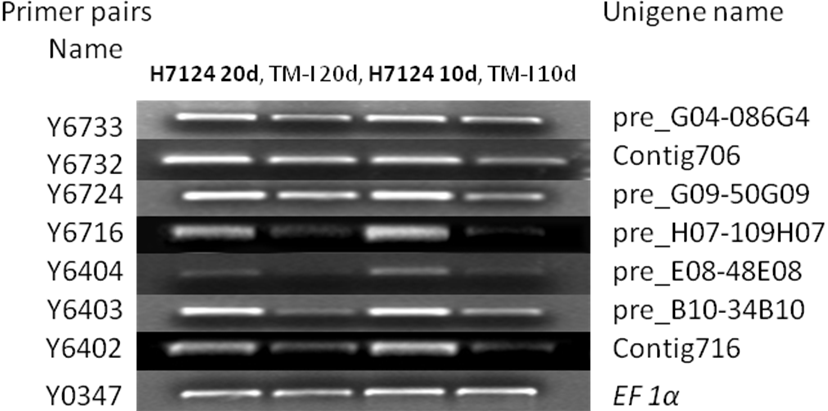
**

**Fig. S1 Experimental verification of candidate unigenes expressed specifically in *G. barbadense* using RT-PCR**

Note: The seven unigenes are selected according high ESTs abundance. 10 and 20 days post anthesis (DPA) fibers cDNA from G. *barbadense* cv. Hai7124 and *G. hirsutum* acc. TM-1, respectively, were used to confirm the expression specificity or predominance in G. *barbadense*. Cotton elongation factor (EF1α) was the internal control for RT-PCR analysis. The primer pairs’ information in RT-PCR analysis was followed as:

| Primer pairs name | Unigene name | Primer pairs information (5'-3') |
| --- | --- | --- |
| Y0347F | EF1α | AGACCACCAAGTACTACTGCAC |
| Y0347R |  | CCACCAATCTTGTACACATCC |
| Y6402F | Contig716 | CGTACAGTGAAAATGAAGTCTCTCCT |
| Y6402R |  | TCCTAAGTAATCGTTGTTGTTCATTG |
| Y6403F | pre_B10-34B10.ab1 | TCGTACAGTGAAAATGAAGTCTCTCC |
| Y6403R |  | CTCTTCCGACTGAATATGATAAAGCC |
| Y6404F | pre_E08-48E08.ab1 | CATGTCTTCCGTTGGTCAACAACC |
| Y6404R |  | CGCGCCATTGGTAATGATTCTACT |
| Y6716F | pre_H07-109H07.ab1 | ATAACAAGCGCGGTGATAG |
| Y6716R |  | AATTCAACCACGCCATTCA |
| Y6724F | pre_G09-50G09.ab1 | GACGGGAAATTTCGCAAGTA |
| Y6724R |  | ACCAATTTCCATCCAGCTCC |
| Y6732F | Contig706 | TAGACAATGAGACAAAGCCACC |
| Y6732R |  | GCTAATCCACGAAAAACGATAC |
| Y6733F | pre_G04-086G4.ab1 | AGATCTTTTCGAGGTCAAGGTC |
| Y6733R |  | TTTAGAGTCAAGGAGGAATGCC |
